# Increased Expression of Proinflammatory Genes in Peripheral Blood Cells Is Associated with Cardiac Cachexia in Patients with Heart Failure with Reduced Ejection Fraction

**DOI:** 10.3390/jcm13030733

**Published:** 2024-01-27

**Authors:** Anja Sandek, Christoph Gertler, Miroslava Valentova, Nadja Jauert, Manuel Wallbach, Wolfram Doehner, Stephan von Haehling, Stefan D. Anker, Jens Fielitz, Hans-Dieter Volk

**Affiliations:** 1 Department of Cardiology and Pneumology, University Medical Center Göttingen, 37075 Göttingen, Germany; christoph.gertler@med.uni-goettingen.de (C.G.); miroslava.valentova@med.uni-goettingen.de (M.V.); stephan.von.haehling@med.uni-goettingen.de (S.v.H.); 2 German Center for Cardiovascular Research (DZHK), Partner Site Göttingen, 37075 Göttingen, Germany; manuel.wallbach@med.uni-goettingen.de; 3 Centre for Stroke Research Berlin, Charité-University Medicine Berlin, Corporate Member of Free University Berlin and Humboldt-University Berlin, 10117 Berlin, Germany; nadja.jauert@charite.de; 4 Division of Physiology, Department of Human Medicine, MSB Medical School Berlin, Rüdesheimerstr 50, 14197 Berlin, Germany; 5 Department of Nephrology and Rheumatology, University Medical Center Göttingen, 37075 Göttingen, Germany; 6 Department of Internal Medicine and Cardiology, Campus Virchow-Klinikum, German Heart Center Charité, Charité-University Medicine Berlin, Corporate Member of Free University Berlin and Humboldt-University Berlin, 13353 Berlin, Germany; wolfram.doehner@charite.de (W.D.); s.anker@cachexia.de (S.D.A.); 7 German Center for Cardiovascular Research (DZHK), Partner Site Berlin, 13353 Berlin, Germany; 8 BIH Center for Regenerative Therapies (BCRT), Charité-University Medicine Berlin, Corporate Member of Free University Berlin and Humboldt-University Berlin, 10117 Berlin, Germany; hans-dieter.volk@charite.de; 9 Department of Internal Medicine B, Cardiology, University Medicine Greifswald, 17475 Greifswald, Germany; jens.fielitz@uni-greifswald.de; 10 German Center for Cardiovascular Research (DZHK), Partner Site Greifswald, 17475 Greifswald, Germany; 11 Department of Medical Immunology, Charité-University Medicine Berlin, Corporate Member of Free University Berlin and Humboldt-University Berlin, 10117 Berlin, Germany

**Keywords:** STAT, SOCS, chronic heart failure, cachexia

## Abstract

**Background**: Cardiac cachexia (CC) in chronic heart failure with reduced ejection fraction (HFrEF) is characterized by catabolism and inflammation predicting poor prognosis. Levels of responsible transcription factors like signal transducer and activator of transcription (STAT)1, STAT3, suppressor of cytokine signaling (SOCS)1 and SOCS3 in peripheral blood cells (PBC) are underinvestigated in CC. Expression of mediators was related to patients’ functional status, body composition (BC) and metabolic gene expression in skeletal muscle (SM). **Methods**: Gene expression was quantified by qRT-PCR in three cohorts: non-cachectic patients (ncCHF, *n* = 19, LVEF 31 ± 7%, BMI 30.2 ± 5.0 kg/m^2^), cachectic patients (cCHF; *n* = 18, LVEF 27 ± 7%, BMI 24.3 ± 2.5 kg/m^2^) and controls (*n* = 17, LVEF 70 ± 7%, BMI 27.6 ± 4.6 kg/m^2^). BC was assessed by dual-energy X-ray absorptiometry. Blood inflammatory markers were measured. We quantified solute carrier family 2 member 4 (SLC2A4) and protein degradation by expressions of proteasome 20S subunit beta 2 and calpain-1 catalytic subunit in SM biopsies. **Results**: TNF and IL-10 expression was higher in cCHF than in ncCHF and controls (all *p* < 0.004). cCHF had a lower fat mass index (FMI) and lower fat-free mass index (FFMI) compared to ncCHF and controls (*p* < 0.05). STAT1 and STAT3 expression was higher in cCHF vs. ncCHF or controls (1.1 [1.6] vs. 0.8 [0.9] vs. 0.9 [1.1] RU and 4.6 [5.5] vs. 2.5 [4.8] vs. 3.0 [4.2] RU, all ANOVA-*p* < 0.05). The same applied for SOCS1 and SOCS3 expression (1.1 [1.5] vs. 0.4 [0.4] vs. 0.4 [0.5] and 0.9 [3.3] vs. 0.4 [1.1] vs. 0.8 [0.9] RU, all ANOVA-*p* < 0.04). In cCHF, higher TNF and STAT1 expression was associated with lower FMI (r = 0.5, *p* = 0.053 and *p* < 0.05) but not with lower FFMI (*p* > 0.4). In ncCHF, neither cytokine nor STAT/SOCS expression was associated with BC (all *p* > 0.3). SLC2A4 was upregulated in SM of cCHF vs. ncCHF (*p* < 0.03). **Conclusions**: Increased STAT1, STAT3, SOCS1 and SOCS3 expression suggests their involvement in CC. In cCHF, higher TNF and STAT-1 expression in PBC were associated with lower FMI. Increased SLC2A4 in cachectic SM biopsies indicates altered glucose metabolism.

## 1. Introduction

Recent advances in understanding the pathophysiology of chronic heart failure (CHF) implicate that CHF is a multi-system disorder that affects both the heart and circulation and the immune, metabolic and musculo-skeletal systems [1,2]. Cardiac cachexia complicates the clinical course in 10–15% of patients because of an anabolic/catabolic dysbalance [3,4] and predicts poor survival in heart failure [5]. The mechanisms of wasting in heart failure are not well understood. High plasma levels of chronically elevated pro-inflammatory cytokines, such as tumor necrosis factor (TNF), are known to be related to cardiac cachexia in advanced heart failure. On a cellular level, the signal transducer and activator of transcription (STAT) mediates the signaling of inflammatory cytokines. Contrastingly, suppressor of cytokine signaling (SOCS) proteins are cytoplasmatic proteins closing a negative feedback loop which reduces certain cytokine signaling that acts via the janus kinase (JAK)/STAT pathway [6]. However, intracellular levels of the transcription factors STAT1, STAT3, SOCS1 and SOCS3 which have been implicated in the wasting process that occurs during aging have not been investigated in cardiac cachexia.

We sought to determine mRNA levels of these immune mediators in peripheral blood cells (PBC) and investigated if their activation is associated with functional parameters, structural alterations of tissue composition and metabolic gene expression in skeletal muscle of HFrEF patients with and without cachexia.

## 2. Methods

We prospectively studied 18 CHF patients with cardiac cachexia (cCHF), 19 patients without cachexia (ncCHF) and 17 control subjects (for demographic and clinical details see Table 1). The diagnosis of CHF with reduced ejection fraction was made when clinical signs and symptoms and left ventricular impairment (left ventricular ejection fraction [LVEF] ≤ 40%) according to guidelines [7] were documented on the day of assessment. Eligibility screening was conducted among all patients in our Wednesday outpatient heart failure clinic. All patients were clinically stable with a medication that did not change within the previous four weeks before assessment. Patients were permitted aspirin at a dosage of 100 mg daily. However, neither antibiotics, steroid hormones nor any non-steroidal anti-inflammatory drugs other than low-dose aspirin were allowed within 4 weeks before examination. CHF medication in cachectic and ncCHF patients is shown in Table 1. None of the control subjects took any medication or supplements on a regular basis at the time of assessment, except for ACE inhibitors and Angiotensin receptor blocking agents each in one subject and beta blockers in two individuals with mild arterial hypertension who did not show left ventricular dysfunction. None of the individuals were competitive athletes and neither cardiopulmonarily nor orthopedically restricted in normal daily activities. Furthermore, they did not use anti-inflammatory drugs such as painkillers such as ibuprofen or aspirin for 3 months prior to the assessments. Subjects with clinical signs of infection, rheumatoid arthritis or history of other autoimmune disorders, renal failure, significant valvular heart disease, intestinal diseases, catabolic conditions such as cancer, change in lifestyle regarding nutritional habits and known metabolic diseases like thyroid dysfunction were excluded. Subjects with immune system disorders or receiving immune modulation therapy were excluded. The study was conducted in accordance with the Declaration of Helsinki and approved by the Institutional Ethics Committee of Charité University Medicine Berlin Campus Virchow (protocol code EA2/71/2004, date of approval 23 June 2004). 

### 2.1. Clinical Assessments

Cachexia was defined as a documented total weight loss of ≥5 percent in 12 months or less according to current consensus-based diagnostic criteria [8]. 

Body mass index (BMI) was calculated as the ratio of weight (kg) and squared height (m^2^). Body composition (fat, lean and bone tissue) was assessed by dual-energy X-ray absorptiometry (DEXA) using a Lunar DPX (Lunar Corp., Madison, WI, USA), as described previously [9], and fat-free mass/height, fat mass/height and bone mass/height were calculated. The measurement accuracy was >98% for lean tissue and >95% for fat tissue. 

Symptom-limited treadmill exercise testing was performed by 93% of subjects with the modified Naughton protocol for assessment of peak oxygen consumption (peak VO_2_), (MedGraphics CPX/D, Medical Graphics Corporation, Cardiorespiratory Diagnostic Systems, St Paul, MN, USA). 

Echocardiography was performed following standard procedures. LVEF was measured using Simpson’s biplane technique. 

### 2.2. RNA Isolation, Reverse Transcription, and qRT-PCR

Blood samples were provided by all subjects after an overnight fast and >10 min of rest in a semi-recumbent position and stored at −80 °C in PAXgeneTM capillary tubes (Qiagen, Hilden, Germany) according to the manufacturer’s protocol for a maximum of 24 months until expression analyses. 

Briefly, the tubes that were stored at −80 °C were centrifuged after thawing for 10 min at 4000 rpm to pellet all blood cells. The gene transcription profile has been obtained from the lysate of sedimented cells from centrifugation of blood aliquots. Total RNA was isolated from the sediment using the Absolutely RNA RT-PCR Miniprep kit (Stratagene, La Jolla, CA, USA) and transcribed into cDNA, as previously described [10]. The cDNA was amplified by quantitative polymerase chain reaction (qRT-PCR) (TaqMan TM, ABI Prism 5700 Sequence Detection System, Applied Biosystems, Foster City, CA, USA) with the use of fluorogenic probes. Samples were tested for genomic DNA contamination. If no gene activity could be detected after 40 PCR cycles or the samples tested positive for genomic DNA, the samples were abolished from further examinations. Gene expression of TNF, STAT1, STAT3, SOCS1, SOCS3, TGFB1 and IL-10 was measured in all but one cCHF patient, one ncCHF patient and two control subjects for STAT1, one cCHF patient, one ncCHF patient and two control subjects for SOCS1 and SOCS3 and one cCHF patient for IL-10. Gene expression was measured in duplicates for each sample.

The sequence of primers used for RT-PCR analysis is shown in the supplement. Annealing for all primers was performed at 60 °C for 30 s. PCR amplification was performed using the ABI Prism 5700 sequence detection system (Applied Biosystems, Foster City, CA, USA). Data were expressed as relative mRNA levels normalized to the stably expressed housekeeping gene hypoxanthine guanine phosphoribosyl transferase (HPRT) with the comparative threshold cycle method (2^−∆∆cT^). All primers and probes were designed, and assays were validated at the Institute of Medical Immunology, Charité University Medicine Berlin, Germany except for the design of STAT1-Primer (Applied Biosystems, Foster City, CA, USA).

### 2.3. Biomarker Measurements

In all participants, venous blood samples were taken in the morning following an overnight fast and after >10 min of rest in a semi-recumbent position. Plasma was collected after centrifugation and frozen at −80 degrees until it was analyzed. 

We measured plasma concentrations of mid-regional pro-adrenomedullin (MR-pro ADM) and mid-regional pro-atrial natriuretic peptide (MR-pro ANP) in a subgroup of 31 patients (19 ncCHF, 13 cCHF) and 15 healthy controls using a sandwich immunoassay (BRAHMS MR-proADM LIA; BRAHMS Aktiengesellschaft, Hennigsdorf, Germany) and using an automated sandwich chemiluminescence immunoassay on the KRYPTOR System (Thermo Fisher/BRAHMS GmbH, Henningsdorf, Germany) as described elsewhere, respectively [11,12]. High-sensitivity C-reactive protein (hsCRP) was detected immunoturbidimetrically. Plasma C-terminal pro-endothelin-1 (CT pro ET-1) was analyzed in a subgroup of 19 patients (14 ncCHF, 5 cCHF) and 14 healthy controls. Therefore, a chemiluminescence sandwich immunoassay was used (CT-proET-1 LIA, BRAHMS GmbH, Hennigsdorf, Germany) as previously described [13]. Plasma concentrations of interleukin 6 (IL-6) we measured in a subgroup of 24 patients (14 ncCHF, 10 cachectic) and 14 healthy controls (Random Access Immunoassay Analyzer, DPC Biermann, Bad Nauheim, Germany).

### 2.4. Cell Stimulation Assays

In a subgroup of 19 patients with CHF (14 ncCHF, 5cCHF) and 12 control subjects, we stimulated fresh whole blood with *Escherichia-coli* (serotype 0111:B4)-derived lipopolysaccharide (LPS). LPS was diluted in Hanks’ balanced salt solution (Sigma-Aldrich, Irvine, UK) and at a concentration of 1 ng/mL was given to the subjects’ blood under sterile conditions. The addition of Hanks’ balanced salt solution alone served as a control. Incubation was performed in a humidified atmosphere (37 °C, 5% CO_2_) for 6 and 24 h, respectively. Supernatants for IL-10 and for TNF content were collected after 6 h (IL-10) or 24 h (TNF), respectively, and stored at −80 °C until analysis (ELISA, R&D Systems, Minneapolis, MN, USA). All samples were analyzed in duplicate and thawed only once for immediate analysis. 

### 2.5. Skeletal Muscle Tissue Biopsy

Percutaneous needle biopsies were obtained from the middle part of the *vastus lateralis* of the quadriceps muscle under local anesthesia in 15 patients with CHF (10 ncCHF, 5cCHF) and 12 healthy control subjects [14]. The biopsies were snap-frozen in liquid nitrogen and stored at −80 °C until analyses. We measured gene expressions of proteasome 20S subunit beta 2 (PSMB2), calpain-1 catalytic subunit (CAPN1) and solute carrier family 2 member 4 (SLC2A4; encoding Glucose Transporter 4 (Glut4)).

### 2.6. Statistical Analysis

StatView 5.0 software (SAS Institute Inc., Cary, NC, USA) was applied to perform all statistical analyses presented below. All data were tested for normality of distribution with the Kolmogorov–Smirnov test. Depending on distribution, data points are reported as means (±SD, compared by unpaired *t*-test) or median (±interquartile range, compared by Mann–Whitney U test). Where applicable, we applied analysis of variance (ANOVA), Student’s unpaired t-test, Fisher´s exact test and Pearson´s simple regression. In sporadic cases indicated in the results, a normal distribution was achieved through log10 transformation of non-normally distributed data. A two-tailed *p*-value ≤ 0.05 was considered significant in all analyses.

## 3. Results

### 3.1. Baseline Characteristics

Clinical data of all patients are provided in Table 1. There were no significant differences in terms of gender and age between heart failure patients and control subjects in the gene expression group (Table 1). Exercise capacity and LVEF were similar in cCHF and ncCHF patients. NYHA class was higher in cCHF compared to ncCHF patients (Table 1).

#### 3.1.1. Body Composition

cCHF patients had both a lower fat mass index (FMI, fat mass per height) and a lower fat-free mass index (FFMI, fat-free mass per height) indicating lower soft lean tissue as compared to ncCHF patients and control subjects (Table 1). Bone mass index (BOMI, bone mass per height) was similar in cCHF patients as compared to ncCHF patients and control subjects (Table 1).

#### 3.1.2. Oxygen Consumption

cCHF patients and ncCHF patients had a similar peak VO_2_ per kg total body weight per minute indicating similar exercise capacity (Table 1). 

### 3.2. Immune Markers

**Figure 1:**
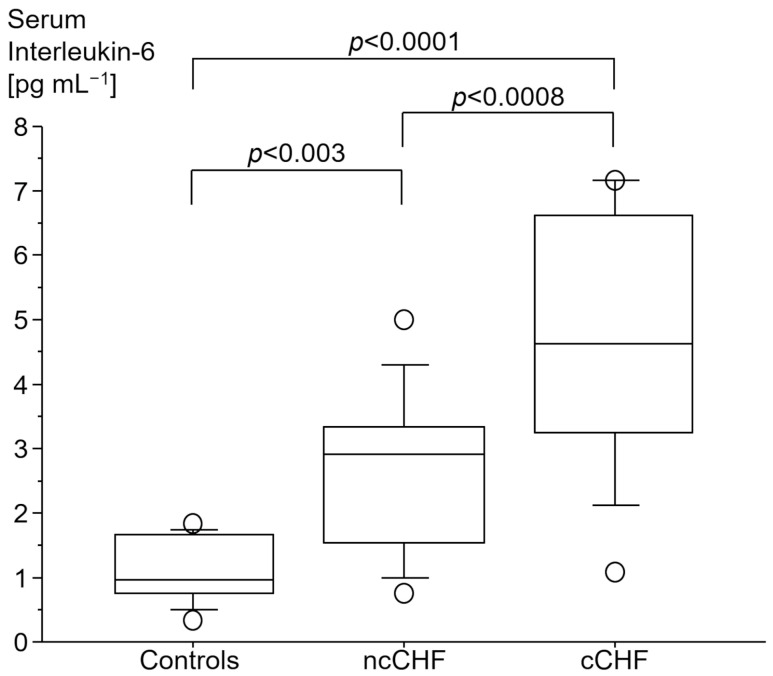
Serum interleukin-6 [pg mL^−1^] in patients with chronic systolic heart failure (CHF) with (cCHF) and without cachexia (ncCHF) and healthy control subjects.

cCHF patients had the highest serum levels of high sensitivity C-reactive protein (hsCRP) as compared to both ncCHF patients and healthy controls (Table 1). There was a gradual increase in plasma IL-6 in cCHF as compared to ncCHF patients or controls with cCHF patients displaying the highest levels (Figure 1).

#### 3.2.1. Cytokine Gene Expression

**Figure 2:**
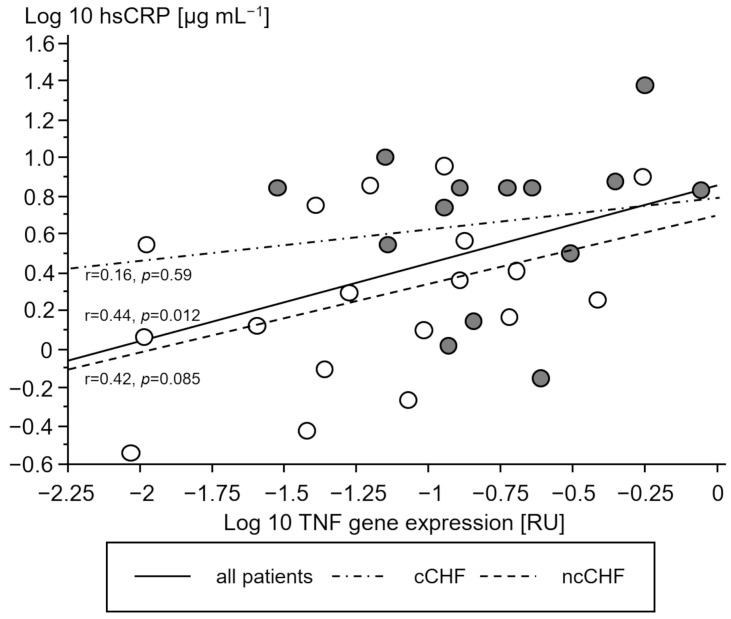
Association between serum high-sensitivity C-reactive protein (hsCRP) [µg mL^−1^] and gene expression of tumor necrosis factor (TNF) in peripheral blood mononuclear cells of chronic systolic heart failure patients (CHF). Open circles represent non-cachectic patients. Closed black circles indicate cachectic patients. Solid line represents the regression of all patients, while the dash-dot line represents regression of cCHF and the dash line the regression of ncCHF patients, respectively.

**Figure 3:**
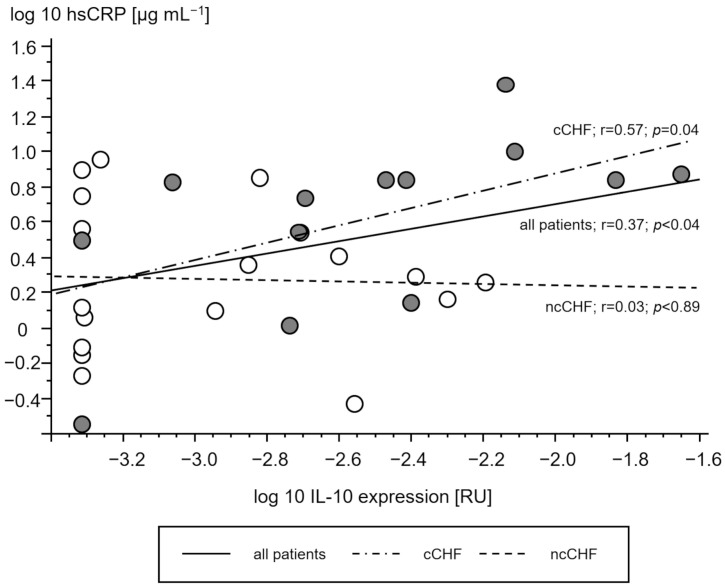
Association of serum high sensitive C reactive protein (hsCRP) [µg/mL] and gene expression of interleukin 10 (IL-10) in peripheral blood mononuclear cells [relative units (RU)] of patients with chronic systolic heart failure. Open circles represent non-cachectic patients. Closed black circles indicate cachectic patients. Solid line represents the regression of all patients, while the dash-dot line represents regression of cCHF and the dash line the regression of ncCHF patients, respectively.

Cachectic patients had a higher TNF- and IL-10 gene expression in PMC as compared to ncCHF patients and controls who showed similar expression (Table 2). A higher gene expression of TNF and IL-10 in cCHF was associated with higher hsCRP in CHF (Figure 2 and Figure 3) but not with plasma IL-6 (*p* > 0.3). TGFB1 expression levels did not differ between groups (*p* > 0.3). However, there was a strong association between higher STAT1 and STAT3 levels in cCHF and ncCHF and healthy controls (r = 0.48, *p* < 0.05).

#### 3.2.2. LPS Stimulation

Upon LPS stimulation ex vivo, the blood of a subgroup of 19 patients with CHF was compared to 12 healthy control subjects. Patients with CHF showed both a higher TNF and a higher IL-10 release as compared to control subjects. Table 3 shows baseline characteristics and results. The limited number of five cCHF patients did not show a higher release from LPS-stimulated whole blood than ncCHF patients (*p* > 0.6). 

#### 3.2.3. Association of STAT and SOCS Genes with Cytokine Gene Expression

In cCHF patients, STAT1-, STAT3-, SOCS1-, SOCS3- expression was higher as compared to ncCHF patients who had levels like controls (Table 2). However, in none of the groups was STAT1-, STAT3-, SOCS1-, SOCS3- expression in PBC directly associated with the level of blood hsCRP or IL-6 levels (Table 4).

In cachectic patients, STAT3 expression was associated with higher TNF expression while STAT1-, SOCS1-, and SOCS3- expression was not (Table 4). Again, STAT3 and on-trend STAT1 expression was associated with higher TGFB1 expression, while SOCS1 and SOCS3 expression was not (Table 4).

In contrast, in ncCHF patients and control subjects, not only STAT1- and STAT3- but also SOCS1- and SOCS3 expression were all associated with both TNF- and TGFB1. Further, in ncCHF but not in cCHF patients, higher on-trend STAT1 expression was associated with a higher IL-10 expression (Table 4). 

In healthy subjects but not in CHF patients, higher SOCS1 expression and on-trend STAT3 and SOCS3 expression were all associated with a higher IL-10 expression. This did not apply to cachectic patients (Table 4). 

#### 3.2.4. Association of Gene Expressions with Body Composition

**Figure 4:**
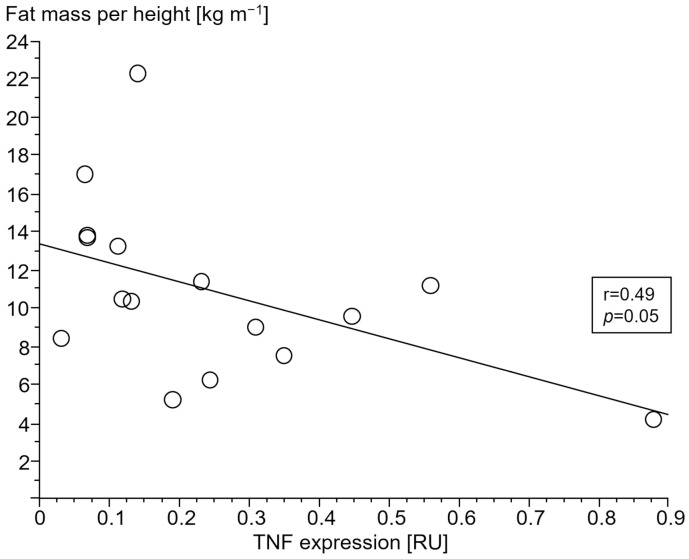
Association of lower fat mass index (kg m^−1^) with tumor necrosis factor (TNF) expression (relative units (RU)) in peripheral blood mononuclear cells of patients with chronic systolic heart failure and cardiac cachexia.

**Figure 5:**
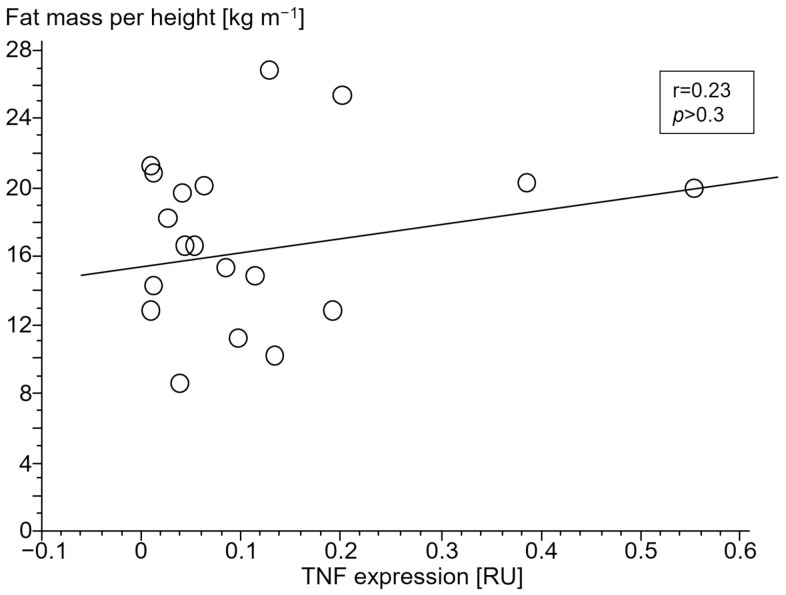
Association of lower fat mass index (kg m^−1^) with tumor necrosis factor (TNF) expression (relative units (RU)) in peripheral blood mononuclear cells of patients with chronic systolic heart failure without cardiac cachexia.

**Figure 6:**
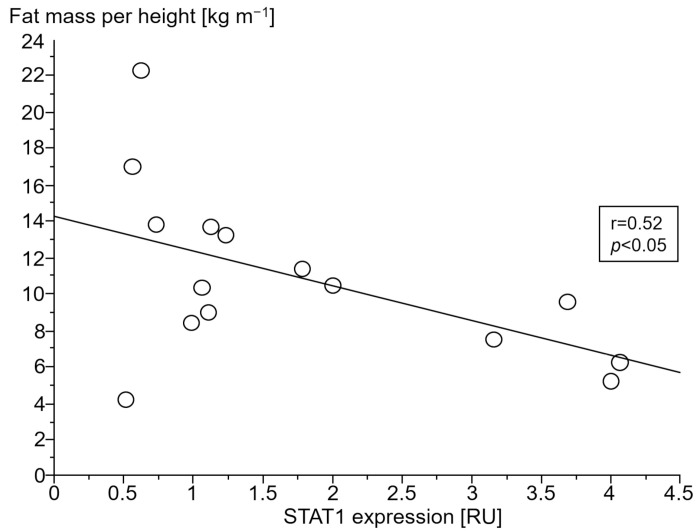
Association between fat mass index (kg m^−1^) and signal transducer and activator of transcription 1 (STAT1) gene expression (relative units (RU)) in peripheral blood mononuclear cells of patients with chronic systolic heart failure and cardiac cachexia.

**Figure 7:**
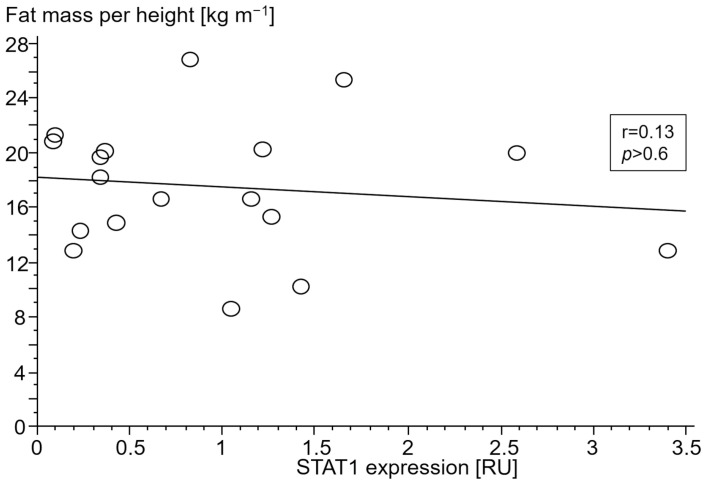
Association between fat mass index (kg m^−1^) and signal transducer and activator of transcription 1 (STAT1) gene expression (relative units (RU)) in peripheral blood mononuclear cells of patients with chronic systolic heart failure without cardiac cachexia.

Only in cCHF patients was lower fat mass index (FMI, fat mass per body height) associated with TNF- and higher STAT1 expression (Figure 4 and Figure 5, for ncCHF: Figure 6 and Figure 7) but not with IL-10 expression (r = 0.09, *p* = 0.7). 

Lower fat-free mass index (FFMI, fat-free mass per body height) was not associated with TNF, STAT1 or IL-10 expression (all *p* > 0.46). 

In ncCHF patients and controls, neither cytokine nor STAT- and SOCS expression in PBC were associated with body composition (all *p* > 0.3).

#### 3.2.5. Association of STAT- and SOCS Expression with Cytokine Release upon LPS Stimulation

In CHF patients, higher TNF- and IL-10-release as well as TNF- and IL-10-release per leucocytes upon LPS stimulation did not show any association with TNF-, IL-10, TGFB1, STAT1, STAT3, SOCS1, or SOCS3 expression in PBC (all *p* > 0.12 and all *p* > 0.28, respectively).

### 3.3. Markers of Oxidative Stress and Vasoconstriction

#### 3.3.1. Mid-Regional Pro-Adrenomedullin

Cachectic patients had the highest levels of mid-regional pro-adrenomedullin (MR-proADM) as compared to nCHF patients and controls (Table 1). MR-proADM was positively associated with IL-10 expression in all CHF patients (r = 0.36, *p* = 0.047). This association in all patients with CHF was triggered by a strong trend in the cCHF subgroup (r = 0.53, *p* = 0.079) and was not significant in ncCHF patients (r = 0.13, *p* = 0.6).

TNF, TGFB1, STAT1, STAT2, SOCS1, and SOCS3 expressions were all not associated with MR-proADM (all *p* > 0.2). 

#### 3.3.2. C-Terminal Pro-Endothelin 1

Cachectic patients had highest levels of C-terminal pro-endothelin 1 (CT-proET-1) as compared to nCHF patients and controls (115 ± 48 vs. 78 ± 23 vs. 60 ± 10 pmol/L, ANOVA *p* < 0.0005). STAT1, STAT2, SOCS1, SOCS3, TNF and IL-10 expression was not associated with CT-proET-1 (*p* > 0.24 for all groups).

### 3.4. Skeletal Muscle Biopsies

#### 3.4.1. Proteasome 20S Subunit Beta 2 (PSMB2)

In trend, a higher PSMB2 expression was found in the skeletal muscle of cCHF as compared to ncCHF patients (Table 5). NcCHF patients and healthy controls had similar expression levels (Table 5).

Both serum IL-6 (*p* > 0.7) and hsCRP (*p* > 0.26) were not associated with higher PSMB2 expression in skeletal muscle neither in cCHF alone nor in all CHF patients.

#### 3.4.2. Calpain-1 Catalytic Subunit (CAPN1)

CAPN1 expression was a trend higher in cCHF as compared to ncCHF patients (Table 5). NcCHF patients and healthy controls showed a similar CAPN1 expression (Table 5).

CAPN1 expression in skeletal muscle was not associated with STAT1 (*p* > 0.55), STAT3 (*p* > 0.6), SOCS1 (*p* > 0.6), SOCS3 (*p* > 0.6), TNF (*p* > 0.3), IL-10 (*p* > 0.9) or TGFB1 (*p* > 0.8) expression in PBC neither in cCHF alone nor in all CHF patients.

Serum IL-6 (*p* > 0.22), TNF (*p* > 0.1) and hsCRP (*p* > 0.37) were not associated with higher CAPN1 expression in skeletal muscle neither in cCHF alone nor in all CHF patients.

#### 3.4.3. Solute Carrier Family 2 Member 4 (SLC2A4)

**Figure 8:**
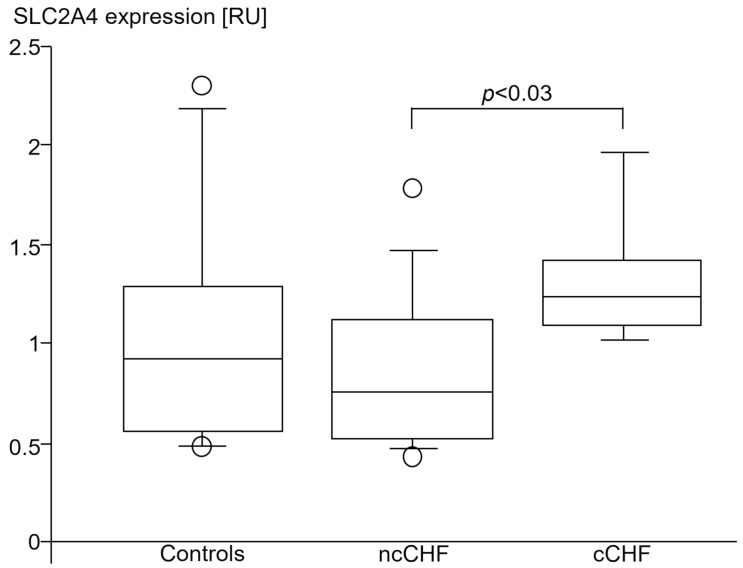
SLC2A4 (GLUT-4) gene expression (relative units (RU)) in *vastus lateralis* skeletal muscle of healthy subjects (controls), patients with chronic systolic heart failure with (cCHF) and without (ncCHF) cardiac cachexia.

**Figure 9:**
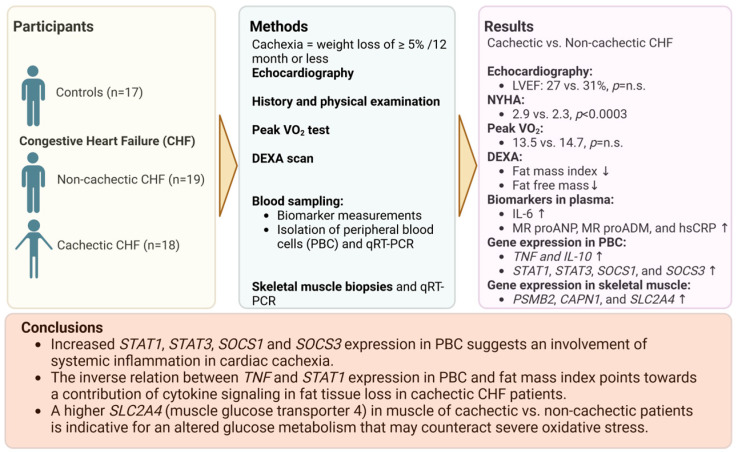
Summary of main results of the study. Created with BioRender.com, accessed on 26 January 2024. N.s. indicates no significance. Arrows pointing up and down represent higher or lower level of the respective factor in cachectic patients.

SLC2A4 expression was higher in skeletal muscle of cCHF compared to ncCHF patients (Table 5, Figure 8, Figure 9). The same applied when only non-diabetic patients with and without cachexia were compared (1.2 [1.1; 1.2] vs. 0.6 [0.5; 0.7], *p* < 0.02).

NcCHF patients and healthy controls had a similar SLC2A4 expression (Table 5, Figure 8). Non-diabetic ncCHF patients showed a lower SLC2A4 expression as compared to healthy controls. However, this did not reach significance (0.6 [0.5; 0.7] vs. 0.9 [0.6; 1.3], *p* > 0.14).

In all CHF patients, SLC2A4 skeletal muscle expression was associated with higher SOCS1 (r = 0.56, *p* < 0.04) and in trend with higher SOCS3 (r = 0.5, *p* = 0.067) and STAT1 (r = 0.47, *p* = 0.08) expression but not with TNF (*p* > 0.3), IL-10 (*p* > 0.4) or TGFB1 (*p* > 0.8) in PBC.

In ncCHF, higher expression of SLC2A4 in skeletal muscle was exclusively associated with STAT1 expression in PBC (r = 0.7, *p* < 0.03). 

In cCHF patients, higher SLC2A4 skeletal muscle expression was associated with SOCS3 (r = 0.9, *p* < 0.03) only and not with SOCS1 (*p* > 0.16), STAT1 (*p* > 0.8), STAT3 (*p* > 0.5), TNF (*p* > 0.3), IL-10 (*p* > 0.7) or TGFB1 (*p* > 0.3) PBC expressions.

Serum hsIL-6 (*p* > 0.1), hsCRP (*p* > 0.15) and hsTNF (*p* > 0.18) were all not associated with higher SLC2A4 skeletal muscle expression neither in cCHF alone nor in all patients with CHF.

### 3.5. Cytokine, STAT and SOCS Expressions and Severity of CHF

In all CHF patients, higher TNF, IL-10, SOCS1 and SOCS3 gene expression was associated with severity of heart failure according to MR-proANP (r = 0.4, *p* < 0.03, r = 0.39, *p* < 0.03, r = 0.45, *p* < 0.03 and r = 0.45, *p* < 0.02, respectively). 

There was no association of TGFB1, STAT1 and STAT3 expression with the severity of heart failure according to blood MR-proANP (*p* > 0.8, *p* > 0.13 and *p* > 0.33).

## 4. Discussion

This is the first study to report an increased STAT1, STAT3, SOCS1 and SOCS3 gene expression in PBC from cCHF patients, which indicates an involvement of the JAK/STAT signaling pathway in cardiac cachexia.

In patients with cardiac cachexia, both higher proinflammatory TNF and higher STAT1 gene expression in PBC were associated with body composition, namely lower fat mass index. In skeletal muscle biopsies from cachectic patients, the expression of the SLC2A4 gene, which encodes for the insulin-dependent glucose transporter 4, was upregulated. In parallel, catabolic function in trend was increased as shown by CAPN1 and PSMB2 as part of the protein-degrading machinery. 

### 4.1. Inflammation Is Increased in Cardiac Cachexia

Catabolic processes in cachexia are commonly governed by proinflammatory cytokines such as TNF. While blood TNF has already been shown to be associated with wasting, we here describe a possible involvement of STAT/SOCS signaling. The fact that lower fat mass in cachectic patients was associated with TNF and STAT1 expression in this study indicates participation of predominantly proinflammatory stimuli. Remarkably, their counter-regulatory partners SOCS1 and SOCS3 were neither associated with TNF nor TGFB1 expression which may indicate an exhaustion of the suppressor of the cytokine signaling system in cCHF. The fact that SOCS1/SOCS3 expression was not associated with lower fat mass index may imply a role of this exhaustion in the development of cardiac cachexia.

### 4.2. Cytokine-Induced Cellular Stress and Damage in the Cachectic Muscle

The cachectic muscle is subject to interlinked cytokine-induced processes that cause damage to the individual myocyte. TNF and IL-10 can induce an imbalance in muscle protein synthesis and degradation in myocytes.

Both cytokines are major inducers of the ubiquitin proteasome system (UPS) which may lead to increased protein degradation in the skeletal muscle as described for cancer cachexia [2]. Our results of the trend of higher proteolytic enzyme expression (i.e., CAPN1, PSMB2) in the cachectic skeletal muscle are in line with this finding. CAPN1 encodes the catalytic subunit of calpain which is involved in the degradation of myofibrillar proteins. This points to muscle wasting due to enhanced proteolysis in our cCHF patients [15,16]. The trend of a higher PSMB2 expression in our cohort is in accord with animal studies on cachectic mice [17] where elevated PSMB2 expression in myocytes resulted in UPS-dependent protein degradation [18]. However, due to the scarce availability of biomaterials, we were not able to investigate if a higher PSMB2 expression was accompanied by an increase in the amount of the PSMB2 protein. Further studies are needed to investigate any changes in the 20S proteasome at mRNA, protein and activity level and to assess if this is associated with muscle wasting and cachexia in CHF patients.

IL-6, on the other hand, is a well-known inducer of mitochondrial damage in myocytes and many other cell types in both human and animal models in case of chronic inflammation [19,20,21,22]. As expected, IL-6 serum concentration was highest in our cCHF patients. In similar settings, i.e., in skeletal muscle [23] and in adipocytes, mitochondria have been observed to appear swollen and show fewer cristae [20]; also, lipid oxidation was shown to be impaired and led to reduced ATP production [20]. Prolonged mitochondrial stress furthermore may lead to a secretion of monocyte chemoattractant protein (MCP), which triggers macrophages to produce further IL-6 and therefore may accelerate or sustain this process [22]. 

### 4.3. SLC2A4 Expression of the Skeletal Muscle

Lower oxygen availability is a common feature of heart failure with critical perfusion during physical exertion. In cCHF patients this can be due to either low perfusion or metabolic stress of skeletal muscle. In fact, we have observed severe oxidative stress in the cCHF group via the proxy marker MR-proADM which was highest in cachectic patients. This oxidative stress furthermore was associated with higher IL-10 expression, which is in accord with studies on rats exposed to hypoxia-induced skeletal muscle injury, where upregulated IL-10 was proven to reduce muscle damage [24]. This was attributed to a restoration of the altered redox equilibrium [25]. Under oxidative stress conditions, anaerobic glycolysis becomes the predominant ATP-producing pathway in skeletal muscle. To compensate for the lower ATP yield, myocytes need to counteract this rising AMP/ATP ratio reflecting energy depletion through stimulation of glucose metabolism. This, in turn, requires a higher glucose uptake [26]. Indeed, a rising AMP/ATP ratio is a well-known mechanism to enhance glucose transporter 4 protein expression, which may explain the higher SLC2A4 expression in the skeletal muscle of cCHF patients. 

Our findings are consistent with previous studies that found mitochondrial damage to increase SLC2A4 expression through AMP-activated protein kinase (AMPK) and associated proteins, which may serve as a rescue mechanism in case of prolonged energy depletion [27]. Energy depletion of skeletal muscle (e.g., due to lower perfusion in CHF) may be further aggravated by the mitochondrial damage during chronic exposure to the high IL-6 observed in our cachectic patients [28]. 

Very much in contrast to the situation in cachectic patients, an inflammation outside a cachexia setting has been described to display an initial downregulation of SLC2A4. 

Isolated inflammation (e.g., by TNF) has been demonstrated to be capable of down-regulating SLC2A4 in both cultivated myocytes and adipocytes [29,30]. 

In line with this, lower expression of corresponding GLUT-4 has been described in non-diabetic non-cachectic patients with CHF-associated inflammation when compared to healthy subjects [31].

In our study on cardiac cachexia, we hypothesize the occurrence of two counteracting effects in human skeletal muscle tissue: first, proinflammatory activation which may decrease SLC2A4 expression [26,29,30], and second, lower perfusion as well as IL-6-conveyed chronic mitochondrial damage of the skeletal muscle. These superposing effects subsequently would lead to energy depletion in the muscle, which may explain the increased SLC2A4 expression in the muscles of cCHF patients. 

In parallel, higher SLC2A4 expression in cCHF skeletal muscle was associated with higher SOCS3 expression in PBC. The latter effect can be explained, as SOCS3 has previously been described to inhibit the unrestricted function of GLUT4 and thus participate in the negative control by preventing further insulin-dependent uptake of glucose into skeletal muscle [32]. 

### 4.4. Lipolysis

Fat mass reduction (e.g., through lipolysis) has previously been described to be facilitated through MR-proANP stimulation of adipocytes [33]. It is thought to enhance energy utilization and thermogenesis through the expression of brown adipocyte-associated genes in adipose tissue [34,35]. ADM is commonly thought to stimulate lipolysis and weight loss in cardiac cachexia [35]. Also, elevated IL-6 levels as measured in our cCHF group are likely to stimulate lipolysis as shown in healthy individuals [36]. Neither MR-proADM nor MR-proANP nor IL-6 showed a direct association with lower fat mass index in our cCHF patients even though they were all elevated in our cCHF patients.

It should be noted, though, that we were able to confirm the well-known association of serum TNF as measured by TNF expression of PBC with lower fat mass index in our study. Expressions of both TNF and STAT1 in PBC were inversely associated with a low fat mass index in cCHF. 

Further studies need to shed more light on the role of the JAK/STAT network in fat cachexia and may pave the way for possible therapeutic applications. 

## 5. Conclusions

In this study, we found an increased proinflammatory pattern of PBC in cachectic CHF patients. Higher STAT1, STAT3, SOCS1 and SOCS3 expression suggests an involvement of this system in inflammatory signaling cascades in cardiac cachexia. While STAT1 expression was associated with lower fat mass index in cCHF patients, SOCS1 and SOCS3 expression was not, which indicates an exhaustion of the counter-regulating suppressor of the cytokine signaling system in cardiac cachexia. The skeletal muscle of cCHF patients showed an altered glucose metabolism with an upregulated SLC2A4 expression. Future studies on larger cohorts may show whether the alterations described here change in the course of disease with aggravating cachexia to shed further insights into the metabolic mechanisms that lead to cardiac cachexia and might therefore be potential therapeutic targets. 

## 6. Limitations

Inflammation and fat wasting are not novel; however, a novelty is that the JAK/STAT system seems to be involved, as suggested by the regression. Increased proinflammatory drive may be either the source, cofactor or result of the body wasting. The origin of the inflammatory activation in the peripheral blood cells cannot be concluded from this study. cCHF patients showed a significantly higher NYHA class compared to ncCHF patients, indicative of a higher degree of heart failure in cCHF patients, which could have affected our analyses. However, both groups did not differ regarding their peak VO_2_ as measured by spiroergometry, which is a more precise tool with significantly less patient-related bias and the current gold standard of stratifying heart failure patients. These results indicate a comparable degree of heart failure in cCHF and ncCHF patients. Additionally, the higher NYHA class reported by cCHF patients might be a result of an increase in peripheral chemosensitivity and severely impaired cardiorespiratory reflex control [37]. Both effects have been described in patients with cachexia as compared to well-matched non-cachectic patients with a degree of impaired cardiorespiratory reflex control more closely related to wasting than to conventional markers of the severity of heart failure [37]. Additionally, wasting diseases also affect the diaphragm and associated muscles of respiration. Hence, one could speculate that higher NYHA classes reported by cCHF patients might at least in part be a result of lower diaphragm and respiratory muscle performance. This well-known phenomenon of respiratory muscle weakness has also been described in 24% of patients with cancer cachexia who proved to be dyspneic, yet free of cardiopulmonary disease [38,39,40]. Additionally, the fact that in cachectic patients there was no correlation of TNF expression and severity of CHF according to MR-proANP indicates an independent association of cachectic state with TNF expression beyond severity in CHF. 

In favor of age-adjusted real-life control patients, we accepted them to be slightly overweight. Older individuals usually tend to gain weight with aging, which we also observed in ncCHF patients who showed a similar BMI in our study. Therefore, our results are only valid for a comparison with slightly overweight, yet plausible control patients of similar age.

The number of patients and control subjects is limited due to the pilot character of the study. Gender distribution was in favor of men, probably due to the higher male prevalence of heart failure in the age group analyzed here.

Due to the scarce availability of biomaterials, our analyses were restricted to quantitation of changes in gene expression. However, it is uncertain if these changes also translate into changes in protein amounts and activity. Further studies are needed to address this important issue. None of the analyzed markers of the JAK/STAT network were associated with the lower FFMI in cachectic patients. Therefore, here, we can propose a role of JAK/STAT only in fat but not in muscle cachexia.

## Figures and Tables

**Table 1:** Baseline data for patients with chronic heart failure (CHF) with (cCHF) and without cachexia (ncCHF) and control subjects.

	Control Subjects (*n* = 17)	ncCHF Patients (*n* = 19)	cCHF Patients (*n* = 18)	cCHF vs. ncCHF Patients *p*-Value	ANOVA-*p*
Number (female)	11(6)	14 (5)	15 (3)	0.5	
NYHA class		2.3 ± 0.5	2.9 ± 0.3	0.0003	
Ejection Fraction (%)	70± 7	31 ± 7 *	27 ± 7 *	0.1	<0.0001
Ischaemic aetiology		13/19	13/18	0.9	
Age (years)	63.4 ± 5.7	67.6 ± 8.8	66.9 ± 10.2	0.8	
Weight (kg)	80.8 ± 14.5	93.9 ± 22.7 *	71.8 ± 9.2	0.0003	0.001
Height (cm)	173.4 ± 9.7	175.6 ± 9.6	171.6 ± 6.0		0.3
BMI (kg/m^2^)	27.5 ± 4.6	30.2 ± 5.0	24.3 ± 2.5 *	0.0002	0.0008
Fat mass/height [kg/m]	14.9 ± 6.6	17.2 ± 4.9 *	10.8 ± 4.5	0.002	0.0077
Fat free mass/height [kg/m]	30.0 ± 5.1	34.4 ± 6.6 *	29.5 ± 3.0	0.008	0.01
Bone mass/height [kg/m]	1.6 ± 0.2	1.6 ± 0.2	1.6 ± 0.2		0.97
Peak VO_2_ (mL/min/kg body weight)	24.9 ± 7.1	14.7 ± 4.6 *	13.5 ± 3.2 *	0.5	<0.0001
Mid regional pro atrial natriuretic peptide (pmol/L)	73 ± 32	165 ± 102 *	341 ± 165 *	*p* < 0.0001	<0.0001
Mid regional pro Adrenomedullin (nmol/L)	0.5 ± 0.1	0.9 ± 0.5 *	1.1 ± 0.7 *	0.08	<0.0001
White Blood Cells (×10^9^/L)	6.6 ± 1.2	7.3 ± 1.6	7.0 ± 1.9		0.4
High sensitive C-reactive Protein (µg/mL)	2.4 ± 3.6	2.9 ± 2.7	6.5 ± 5.8 *	*p* < 0.05	<0.006
Beta blocker		84%	94%	0.3	
Angiotensin converting enzyme (ACE) inhibitor		74%	56%	0.4	
AT_1_ receptor blocker (ARB)		32%	39%	0.6	
Mineralocorticoid receptor antagonist		53%	83%	0.08	
Loop diuretic		63%	89%	0.2	
Statin		74%	83%	0.7	

Results are presented as mean ± standard deviation or median (interquartile range) as appropriate. * *p* < 0.05 vs. control subjects.

**Table 2:** Gene expression in peripheral blood mononuclear cells of patients with chronic systolic heart failure (CHF) with (cCHF) and without (ncCHF) cachexia.

Gene Expression [RU ^+^]	Control Subjects (*n* = 17)	All CHF Patients (*n* = 36)	ncCHF Patients (*n* = 19)	cCHF Patients (*n* = 18)	*p*-Value cCHF vs. ncCHF	ANOVA-*p*
*STAT1*	0.97 ± 0.76	1.30 ± 1.13	0.96 ± 0.90	1.67 ± 1.26 *	<0.03	<0.05
*STAT3*	3.70 ± 2.49	4.40 ± 3.14	3.63 ± 3.03	5.20 ± 3.12	<0.05	0.07
*SOCS1*	0.47 ± 0.30	0.88 ± 0.68	0.50 ± 0.32	1.22 ± 0.73 *	<0.0003	<0.0002
*SOCS3*	1.0 ± 0.92	1.80 ± 2.80	0.90 ±1.46	2.51 ± 3.51 *	<0.01	<0.03
*TNF*	0.12 ± 0.10	0.20 ± 0.24	0.12 ± 0.14	0.28 ± 0.30 *	<0.004	<0.009
*IL10*	0.002 ± 0.004	0.003 ± 0.005	0.002 ± 0.002	0.005 ± 0.006 *	<0.008	<0.02
*TGFB1*	88.9 ± 63.8	85.6 ± 60.6	85.4 ± 74.3	83.4 ± 47.7	0.2	0.4

STAT1 indicates signal transducer and activator of transcription 1; STAT3, signal transducer and activator of transcription 3; SOCS1, suppressor of cytokine signaling 1; SOCS3, suppressor of cytokine signaling 3; TNF, tumor necrosis factor; IL10, interleukin 10; TGFB1, transforming growth factor beta 1. Results are presented as mean ± standard deviation. ^+^ RU = relative units; * *p* < 0.05 vs. control subjects; *p*-values for subgroup comparisons are provided if ANOVA *p* < 0.05, with the exception of STAT-3 comparison (ANOVA-*p* = 0.07).

**Table 3:** Tumor necrosis factor (TNF) and interleukin 10 (IL-10) in fresh whole blood after stimulation by *Escherichia-coli* (serotype 0111:B4)-derived lipopolysaccharide for 6 (TNF) and 24 (IL-10) hours of patients with chronic heart failure (CHF) with (cCHF) and without cachexia (ncCHF) and control subjects. Results are presented as mean ± standard deviation. *p*-values are given for the comparison of total CHF patients and control subjects.

	Control Subjects (*n* = 12)	All CHF Patients (19)	ncCHF Patients (*n* = 14)	cCHF Patients (*n* = 5)	All CHF Patients vs. Controls *p*-Value
Stimulated tumor necrosis factor per blood cells [pg × 10^−6^]	2.26 ± 1.18	4.41 ± 2.34	4.31 ± 2.30	4.64 ± 2.69	0.007
Stimulated interleukin 10 per blood cells [pg × 10^−6^]	0.17 ± 0.12	0.38 ± 0.28	0.35 ± 0.24	0.43 ± 0.38	0.02
White blood Cells (×10^9^/L)	6.7 ± 1.1	7.6 ± 1.6	7.5 ± 1.6	8.0 ± 1.6	0.07
Red blood cells (×10^9^/L)	4.5 ± 0.4	4.4 ± 0.5	4.4 ± 0.5	4.2 ± 0.5	0.6
Blood thrombocytes (×10^9^/L)	228 ± 39	222 ± 85	232 ± 97	199 ± 48	0.6
Number (female)	6 (6)	15 (4)	10 (4)	5 (0)	0.13
Age (years)	66 ± 4	68 ± 9	67 ± 9	72 ± 10	0.3
NYHA class		2.3 ± 0.5	2.1 ± 0.4	2.8 ± 0.4	
Ejection Fraction (%)	71 ± 7	31 ± 7	32 ± 6	28 ± 8	
Ischaemic aetiology		14/19	10/14	4/5	
BMI (kg/m^2^)	28 ± 5	28 ± 5	29 ± 5	24 ± 2	0.8
Fat mass/height [kg/m]	16.4 ± 6.3	14.7 ± 5.7	16.5 ± 5.0	9.8 ± 2.6	0.4
Fat-free mass/height [kg/m]	29.2 ± 5.4	32.6 ± 6.0	33.6 ± 6.7	29.8 ± 1.9	0.13
Peak VO_2_ (mL/min/kg body weight)	22.6 ± 4.2	15.0 ± 4.5	15.9 ± 4.3	12.1 ± 4.2	<0.0001
Mid regional pro atrial natriuretic peptide (pmol/L)	75 ± 35	176 ± 113	130 ± 68	304 ± 121	0.006
Mid regional pro Adrenomedullin (nmol/L)	0.5 ± 0.8	0.8 ± 0.3	0.7 ± 0.3	1.0 ± 0.3	0.005
High sensitive C-reactive Protein (µg/mL)	3.3 ± 4.0	3.7 ± 5.4	2.1 ± 2.0	8.2 ± 9.3	0.8
Beta blocker		89%	86%	100%	
Angiotensin converting enzyme (ACE) inhibitor		74%	71%	80%	
AT_1_ receptor blocker (ARB)		32%	36%	20%	
Mineralocorticoid receptor antagonist		68%	64%	80%	
Loop diuretic		58%	50%	80%	
Statin		74%	71%	80%	

Results are presented as mean ± standard deviation.

**Table 4:** Association of STAT and SOCS gene expression with cytokine gene expression in peripheral blood mononuclear cells of patients with chronic systolic heart failure (CHF) with (cCHF) and without (ncCHF) cachexia and control subjects. *p*-values (significant *p*-values in bold) and correlation coefficients r are given.

		Gene Expression			
		STAT1	STAT3	SOCS1	SOCS3
Controls	blood hsCRP levels	*p* = 0.4, r = 0.1	*p* = 0.6, r = 0.1	*p* = 0.5, r = 0.2	*p* = 0.6, r = 0.2
	blood IL-6 levels	*p* = 0.5, r = 0.2	*p* = 0.6, r = 0.2	*p* = 1.0, r = 0.01	*p* = 0.4, r = 0.3
	TNF expression	*p* = 0.0005, r = 0.8	*p* < 0.0001, r = 0.9	*p* < 0.0001, r = 0.9	*p* < 0.0001, r = 0.9
	IL-10 expression	*p* = 0.1, r = 0.4	*p* = 0.051, r = 0.5	*p* = 0.02, r = 0.6	*p* < 0.0001, r = 0.9
	TGFβ1 expression	*p* < 0.0001, r = 0.8	*p* < 0.0001, r = 1.0	*p* < 0.0001, r = 0.8	*p* < 0.0001, r = 0.9
ncCHF	blood hsCRP levels	*p* = 0.8, r = 0.1	*p* = 0.8, r = 0.1	*p* = 0.14, r = 0.4	*p* = 0.5, r = 0.2
	blood IL-6 levels	*p* = 1.0, r = 0.01	*p* = 0.7, r = 0.1	*p* = 0.2, r = 0.4	*p* = 0.6, r = 0.2
	TNF expression	*p* < 0.0001, r = 0.9	*p* = 0.0002, r = 0.8	*p* < 0.0001, r = 0.8	*p* < 0.0001, r = 0.9
	IL-10 expression	*p* = 0.054, r = 0.5	*p* = 0.1, r = 0.4	*p* = 0.2, r = 0.3	*p* < 0.0001, r = 0.9
	TGFβ1 expression	*p* = 0.001, r = 0.7	*p* = 0.0002, r = 0.7	*p* = 0.007, r = 0.6	*p* = 0.0002, r = 0.7
cCHF	blood hsCRP levels	*p* = 0.5, r = 0.2	*p* = 0.3, r = 0.3	*p* = 0.4, r = 0.3	*p* = 0.8, r = 0.1
	blood IL-6 levels	*p* = 0.2, r = 0.5	*p* = 0.4, r = 0.3	*p* = 0.3, r = 0.4	*p* = 0.1, r = 0.6
	TNF expression	*p* = 0.4, r = 0.2	*p* = 0.01, r = 0.6	*p* = 0.4, r = 0.2	*p* = 0.6, r = 0.1
	IL-10 expression	*p* = 0.5, r = 0.2	*p* = 0.5, r = 0.2	*p* = 0.5, r = 0.2	*p* = 0.6, r = 0.1
	TGFβ1 expression	*p* = 0.06, r = 0.5	*p* < 0.0001, r = 0.9	*p* = 0.4, r = 0.2	*p* = 0.9, r = 0.03

**Table 5:** Skeletal muscle expression of Proteasome 20S subunit beta 2, Calpain-1 catalytic subunit and solute carrier family 2 member 4 (encoding Glucose Transporter 4) in patients with chronic heart failure (CHF) with (cCHF) and without cachexia (ncCHF) and control subjects.

	Control Subjects (*n* = 12)	ncCHF Patients (*n* = 10)	cCHF Patients (*n* = 5)	cCHF vs. ncCHF Patients *p*-Value
Proteasome 20S subunit beta 2	1.0 ± 0.6	0.7 ± 0.2	0.9 ± 0.2	0.086
Calpain-1 catalytic subunit	0.6 ± 0.4	0.7 ± 0.3	1.1 ± 0.5	0.086
Solute carrier family 2 member 4	1.1 ± 0.6	0.8 ± 0.4	1.3 ± 0.4	<0.03

Results are presented as mean ± standard deviation. PSMB2 expression in skeletal muscle was a trend associated with serum TNF in cCHF (r = 0.9, *p* = 0.07). It was not associated with STAT1 (*p* > 0.05), STAT3 (*p* > 0.07), SOCS1 (*p* > 0.5), SOC S3 (*p* > 0.8), TNF (*p* > 0.19), IL-10 (*p* > 0.6) or TGFB1 (*p* > 0.09, r= 0.8, all CHF *p* > 0.7) PBC expression neither in cCHF alone nor in all CHF patients.

## Data Availability

The original contributions presented in the study are included in the article, further inquiries can be directed to the corresponding authors.

## Abbreviations

CAPN1	calpain-1 catalytic subunit
CHF	chronic heart failure
cCHF	chronic heart failure patients with cachexia
Glucose Transporter 4	Glut4
hs	high sensitivity
ncCHF	chronic heart failure patients without cachexia
Peak VO_2_	maximum oxygen consumption
LVEF	left ventricular ejection fraction
TNF	tumor necrosis factor
TGF	transforming growth factor
IL	interleukin
TNF	tumor necrosis factor
JAK	janus (tandem) kinase
PBC	peripheral blood cells
STAT	signal transducer and activator of transcription
SOCS	suppressors of cytokine signaling
PSMB2	proteasome 20S subunit beta 2
SLC2A4	solute carrier family 2 member 4

## References

[1] Philippou A., Xanthis D., Chryssanthopοulos C., Maridaki M., Koutsilieris M. (2020). Heart Failure-Induced Skeletal Muscle Wasting. Curr. Heart Fail. Rep..

[2] Levine B., Kalman J., Mayer L., Fillit H.M., Packer M. (1990). Elevated circulating levels of tumor necrosis factor in severe chronic heart failure. N. Engl. J. Med..

[3] Josiak K., Jankowska E.A., Piepoli M.F., Banasiak W., Ponikowski P. (2014). Skeletal myopathy in patients with chronic heart failure: Significance of anabolic-androgenic hormones. J. Cachexia Sarcopenia Muscle.

[4] Nagaya N., Uematsu M., Kojima M., Date Y., Nakazato M., Okumura H., Hosoda H., Shimizu W., Yamagishi M., Oya H. (2001). Elevated circulating level of ghrelin in cachexia associated with chronic heart failure: Relationships between ghrelin and anabolic/catabolic factors. Circulation.

[5] Rahman A., Jafry S., Jeejeebhoy K., Nagpal A.D., Pisani B., Agarwala R. (2016). Malnutrition and Cachexia in Heart Failure. JPEN J. Parenter. Enteral Nutr..

[6] Matsukawa A. (2007). STAT proteins in innate immunity during sepsis: Lessons from gene knockout mice. Acta Med. Okayama.

[7] Ponikowski P., Voors A.A., Anker S.D., Bueno H., Cleland J.G.F., Coats A.J.S., Falk V., González-Juanatey J.R., Harjola V.P., Jankowska E.A. (2016). 2016 ESC Guidelines for the diagnosis and treatment of acute and chronic heart failure: The Task Force for the diagnosis and treatment of acute and chronic heart failure of the European Society of Cardiology (ESC) Developed with the special contribution of the Heart Failure Association (HFA) of the ESC. Eur. Heart J..

[8] Evans W.J., Morley J.E., Argilés J., Bales C., Baracos V., Guttridge D., Jatoi A., Kalantar-Zadeh K., Lochs H., Mantovani G. (2008). Cachexia: A new definition. Clin. Nutr..

[9] Riggio O., Andreoli A., Diana F., Fiore P., Meddi P., Lionetti R., Montagnese F., Merli M., Capocaccia L., De Lorenzo A. (1997). Whole body and regional body composition analysis by dual-energy X-ray absorptiometry in cirrhotic patients. Eur. J. Clin. Nutr..

[10] Platzer C., Ode-Hakim S., Reinke P., Docke W.D., Ewert R. (1994). Volk HD: Quantitative PCR analysis of cytokine transcription patterns in peripheral mononuclear cells after anti-CD3 rejection therapy using two novel multispecific competitor fragments. Transplantation.

[11] Morgenthaler N.G., Struck J., Alonso C., Bergmann A. (2005). Measurement of midregional proadrenomedullin in plasma with an immunoluminometric assay. Clin. Chem..

[12] Maisel A., Mueller C., Nowak R., Peacock W.F., Landsberg J.W., Ponikowski P., Mockel M., Hogan C., Wu A.H., Richards M. (2010). Mid-region pro-hormone markers for diagnosis and prognosis in acute dyspnea: Results from the BACH (Biomarkers in Acute Heart Failure) trial. J. Am. Coll. Cardiol..

[13] Ferro C.J., Spratt J.C., Haynes W.G., Webb D.J. (1998). Inhibition of neutral endopeptidase causes vasoconstriction of human resistance vessels in vivo. Circulation.

[14] Bergstrom J. (1975). Percutaneous needle biopsy of skeletal muscle in physiological and clinical research. Scand. J. Clin. Lab. Investig..

[15] Oliver W.T., Keel B.N., Lindholm-Perry A.K., Horodyska J., Foote A.P. (2018). The effects of Capn1 gene inactivation on the differential expression of genes in skeletal muscle. Gene.

[16] Costelli P., De Tullio R., Baccino F.M., Melloni E. (2001). Activation of Ca(2+)-dependent proteolysis in skeletal muscle and heart in cancer cachexia. Br. J. Cancer.

[17] Todorov P., Cariuk P., McDevitt T., Coles B., Fearon K., Tisdale M. (1996). Characterization of a cancer cachectic factor. Nature.

[18] Wyke S.M., Tisdale M.J. (2005). NF-kappaB mediates proteolysis-inducing factor induced protein degradation and expression of the ubiquitin-proteasome system in skeletal muscle. Br. J. Cancer.

[19] Skuratovskaia D., Komar A., Vulf M., Quang H.V., Shunkin E., Volkova L., Gazatova N., Zatolokin P., Litvinova L. (2021). IL-6 Reduces Mitochondrial Replication, and IL-6 Receptors Reduce Chronic Inflammation in NAFLD and Type 2 Diabetes. Int. J. Mol. Sci..

[20] Ji C., Chen X., Gao C., Jiao L., Wang J., Xu G., Fu H., Guo X., Zhao Y. (2011). IL-6 induces lipolysis and mitochondrial dysfunction, but does not affect insulin-mediated glucose transport in 3T3-L1 adipocytes. J. Bioenerg. Biomembr..

[21] Abid H., Ryan Z.C., Delmotte P., Sieck G.C., Lanza I.R. (2020). Extramyocellular interleukin-6 influences skeletal muscle mitochondrial physiology through canonical JAK/STAT signaling pathways. FASEB J..

[22] Qualls A.E., Southern W.M., Call J.A. (2021). Mitochondria-cytokine crosstalk following skeletal muscle injury and disuse: A mini-review. Am. J. Physiol-Cell Physiol..

[23] Carson J.A., Hardee J.P., VanderVeen B.N. (2016). The emerging role of skeletal muscle oxidative metabolism as a biological target and cellular regulator of cancer-induced muscle wasting. Semin. Cell Dev. Biol..

[24] Ozturk K., Demir B., Oke R., Durmaz H. (2006). Dose-related effects of recombinant human interleukin-10 on hypoxia-induced skeletal muscle injury in immature rats. J. Orthop. Sci..

[25] Latorre E., Matheus N., Layunta E., Alcalde A.I., Mesonero J.E. (2014). IL-10 counteracts proinflammatory mediator evoked oxidative stress in Caco-2 cells. Mediat. Inflamm..

[26] Huang S., Czech M.P. (2007). The GLUT4 glucose transporter. Cell Metab..

[27] Ke R., Xu Q., Li C., Luo L., Huang D. (2018). Mechanisms of AMPK in the maintenance of ATP balance during energy metabolism. Cell Biol. Int..

[28] Lv J., Li Y., Shi S., Xu X., Wu H., Zhang B., Song Q. (2022). Skeletal muscle mitochondrial remodeling in heart failure: An update on mechanisms and therapeutic opportunities. Biomed. Pharmacother..

[29] Furuya D.T., Neri E.A., Poletto A.C., Anhê G.F., Freitas H.S., Campello R.S., Rebouças N.A., Machado U.F. (2013). Identification of nuclear factor-κB sites in the Slc2a4 gene promoter. Mol. Cell Endocrinol..

[30] Ebersbach-Silva P., Poletto A.C., David-Silva A., Seraphim P.M., Anhê G.F., Passarelli M., Furuya D.T., Machado U.F. (2018). Palmitate-induced Slc2a4/GLUT4 downregulation in L6 muscle cells: Evidence of inflammatory and endoplasmic reticulum stress involvement. Lipids Health Dis..

[31] Doehner W., Gathercole D., Cicoira M., Krack A., Coats A.J., Camici P.G., Anker S.D. (2010). Reduced glucose transporter GLUT4 in skeletal muscle predicts insulin resistance in non-diabetic chronic heart failure patients independently of body composition. Int. J. Cardiol..

[32] Calegari V.C., Alves M., Picardi P.K., Inoue R.Y., Franchini K.G., Saad M.J., Velloso L.A. (2005). Suppressor of cytokine signaling-3 Provides a novel interface in the cross-talk between angiotensin II and insulin signaling systems. Endocrinology.

[33] Kalra P.R., Tigas S. (2002). Regulation of lipolysis: Natriuretic peptides and the development of cachexia. Int. J. Cardiol..

[34] Bordicchia M., Liu D., Amri E.Z., Ailhaud G., Dessì-Fulgheri P., Zhang C., Takahashi N., Sarzani R., Collins S. (2012). Cardiac natriuretic peptides act via p38 MAPK to induce the brown fat thermogenic program in mouse and human adipocytes. J. Clin. Investig..

[35] Christensen H.M., Kistorp C., Schou M., Keller N., Zerahn B., Frystyk J., Flyvbjerg A., Faber J. (2014). Cross-talk between the heart and adipose tissue in cachectic heart failure patients with respect to alterations in body composition: A prospective study. Metabolism.

[36] Huang Q., Wu M., Wu X., Zhang Y., Xia Y. (2022). Muscle-to-tumor crosstalk: The effect of exercise-induced myokine on cancer progression. Biochim. Biophys. Acta Rev. Cancer.

[37] Ponikowski P., Piepoli M., Chua T.P., Banasiak W., Francis D., Anker S.D., Coats A.J. (1999). The impact of cachexia on cardiorespiratory reflex control in chronic heart failure. Eur. Heart J..

[38] Ripamonti C., Bruera E. (1997). Dyspnea: Pathophysiology and assessment. J. Pain Symptom Manag..

[39] Dudgeon D., Baracos V.E. (2016). Physiological and functional failure in chronic obstructive pulmonary disease, congestive heart failure and cancer: A debilitating intersection of sarcopenia, cachexia and breathlessness. Curr. Opin. Support. Palliat. Care.

[40] Coats A.J. (2002). Origin of symptoms in patients with cachexia with special reference to weakness and shortness of breath. Int. J. Cardiol..

